# Psychological wellbeing among parents of a child living with a serious chronic illness: A cross-sectional survey study

**DOI:** 10.1177/13674935241238485

**Published:** 2024-03-29

**Authors:** Eden G Robertson, Lauren Kelada, Robert Ilin, Elizabeth Emma Palmer, Ann Bye, Adam Jaffe, Sean E Kennedy, Chee Y Ooi, Donna Drew, Claire E Wakefield

**Affiliations:** 1 Discipline of Paediatrics and Child Health, School of Clinical Medicine, UNSW Medicine and Health, 7800 UNSW Sydney, Randwick, NSW, Australia; 2 Behavioural Sciences Unit, Kids Cancer Centre, Sydney Children’s Hospital, Randwick, NSW, Australia; 3 Centre for Clinical Genetics, Sydney Children’s Hospitals Network, Randwick, NSW, Australia; 4 Department of Neurology, Sydney Children’s Hospital, Randwick, NSW, Australia; 5 Department of Respiratory Medicine, Sydney Children’s Hospital, Randwick, NSW, Australia; 6 Department of Nephrology, Sydney Children’s Hospital, Randwick, NSW, Australia; 7 Department of Gastroenterology, Sydney Children’s Hospital, Randwick, NSW, Australia; 8 Kids Cancer Centre, Sydney Children’s Hospital, Randwick, NSW, Australia

**Keywords:** Caregivers, child health, chronic disease, psychological distress, psychosocial functioning

## Abstract

Parents of a child with a chronic illness can experience greater distress than the average population, yet little is understood about differences between illness groups. This cross-sectional survey study aimed to compare parents’ psychological distress and perceived wellbeing across five chronic illnesses. Parents from one Australian pediatric hospital completed the Kessler Psychological Distress Scale and seven purpose-designed items about their wellbeing. Data from 106 parents (cancer = 48, cystic fibrosis [CF] = 27, kidney disease = 12, gastrointestinal condition/disorder = 9, developmental and epileptic encephalopathy [DEE] = 10) was analysed using bivariate Pearson’s Correlation and linear mixed-effects models. Parents’ distress scores differed between groups (*F*(4,80) = 2.50, *p* = .049), with the DEE group reporting higher distress than the CF group (*mean difference* = 6.76, 95% CI [0.11, 13.42]). Distress scores were moderately correlated to parents’ perceptions of their child’s health and their own wellbeing. Parents’ self-reported coping with their child’s condition/treatments differed (*F*(4,81) = 3.24, *p* = .016), with the DEE group rating their coping as poorer than the CF group (*mean difference* = −25.32, 95% CI [−46.52, 4.11]). Across all groups, parents reported unmet needs, particularly for psychosocial support and practical/financial assistance. Support interventions may be most effective if tailored to the child’s illness, with greater support potentially needed for parents who have a child with DEE and/or severe comorbidities.

## Introduction

In Western countries, childhood chronic illness occurs in 10%–25% of children under 18 years of age (Australian Institute of Health and Welfare, 2020). Children often have several complex comorbidities (Australian Institute of Health and Welfare, 2020) and encounter frequent hospitalisations, absences from school, and challenging treatment regimens (Fleming et al., 2019; Lum et al., 2019). The responsibility of day-to-day care falls to parents in most cases, which they must balance with other parental and employment responsibilities, as well as their social life and relationships, and personal aspirations (Kish et al., 2018).

Given the challenges that can arise in caring for a child living with a serious chronic illness, parents frequently report higher distress and poorer mental health than peers, including clinically relevant anxiety and depression (Cohn et al., 2020; Kelada et al., 2020a; Rosenberg et al., 2013). Parents with a child with a chronic illness that is also at high risk for behaviour problems, such as children with developmental delay, may report even greater psychological distress than parents of children with chronic illness without behaviour problems (Barroso et al., 2018). Prior research suggests that parental distress may differ according to illness-specific characteristics of their child, such as severity and chronicity (Spratt et al., 2007). However, there is minimal research comparing distress across different disease groups. This data is necessary as it will inform the development of psychosocial interventions for families most in need, and that target aspects of their child’s condition which contributes most to their distress.

Research has commonly investigated parental wellbeing within the context of childhood cancer (Bakula et al., 2020; Bruce, 2006). Fewer studies have examined parental wellbeing in rare illnesses (Manalel et al., 2022; Pelentsov et al., 2015). Families of a child with a rare disease may experience unique challenges with the under-recognition of their child’s condition, such as lack of information and support, and potentially a lengthy ‘diagnostic odyssey’ (i.e. the time between onset of symptoms and final diagnosis) (Bauskis et al., 2022; Robertson et al., 2023). Current evidence suggests these challenges cause significant distress to families (Forman et al., 2012; Nevin et al., 2020b, 2022). Parents of children with rare and under-recognised diseases may therefore be particularly vulnerable to experiencing poor mental health. However, lack of research into the mental health impacts of rare disease on families means that targeted mental health services are severely lacking (Nevin et al., 2020a; Villas et al., 2017; Rare Voices Australia, 2020). Research is needed to build the evidence for further clinical and policy investment in services for these vulnerable families.

Our current study addresses the identified research gaps by comparing psychological wellbeing and support needs among parents of children with five serious illnesses. To ensure that we are providing the most appropriate support resources to families, it is critical that we better understand parental distress and any illness-specific support needs. The information garnered from this study will contribute to the provision of more support tailored to the needs of parents of children from different illness groups.

### Aim

1. The primary aim is to explore how parents report their own distress, and their own and their child’s wellbeing, and whether this differs across illness groups.
2. The secondary aim is to identify parents describe their support needs, and whether this differs across illness groups.

## Method

The current study was part of a larger study, ‘SibStars’, a cross-sectional survey study investigating the wellbeing of parents and siblings of young people with chronic illness (Kelada et al., 2022). As such, parents were only eligible to participate if they had a child with a chronic illness and that least one healthy sibling. We received ethical approval for this study from the Sydney Children’s Hospitals Network Human Research Ethics Committee (LNR/18/SCHN/449) and carried out our study in accordance with the Helsinki Declaration of the World Medical Association.

### Inclusion and exclusion criteria

We invited parents to participate in SibStars between April, 2019 and April, 2020. Parents were eligible to participate in SibStars if they had (i) a child (<18 years of age) with a chronic illness and were diagnosed ≥6 months prior to study invite; and (ii) at least one ‘healthy’ child still living at home. Parents were required to provide informed consent and be able to speak and read conversational English. Parents were ineligible if they had insufficient English language skills to complete the initial intake and/or understand the core aims of the study, their child with chronic illness was diagnosed less than 6 months prior to the study, and if their child with a chronic illness had died, or their child was in end-of-life care. More than one parent per family could participate. We actively focused recruitment on five diagnoses: cancer (either on or off-treatment), cystic fibrosis (CF), chronic kidney disease (herein ‘kidney disease’), a chronic gastrointestinal condition/disorder (herein ‘gastrointestinal disorder’), and developmental and epileptic encephalopathy (DEE). We selected these diagnoses primarily based on the admission rates at our recruiting hospital.

### Procedure

We used convenience and purposive sampling to recruit for our study. Clinicians provided the contact details of eligible families to the SibStars team and/or invited the SibStars team to recruit in clinic. During initial contact (email, phone, or face-to-face), our team discussed the study purpose and then followed up with parents who expressed interest by emailing or mailing them an information sheet and consent form, a questionnaire (and reply-paid envelope for mailed information). For recruitment purposes only, we used the Distress Thermometer (Mitchell et al., 2010) to indicate whether parents were highly distressed and in need of further support. Five parents met our defined cutoff of ≥8 out of 10 in their completed questionnaire. A trained psychosocial researcher called these parents to assess their mood, determine their available supports, and to refer them to external support services if necessary.

### Measures

As part of the larger SibStars questionnaire, we collected demographics and the child’s medical characteristics, and parents’ level of distress via the Kessler Psychological Distress (K10) Scale (Andrews and Slade, 2001), and perceived wellbeing, for themselves and their child via purpose-designed Visual Analogue Scales (VAS). We report on only these measures for this paper (see Table 1 for more details on measures being reported on).

**Table 1.** Description of measures/items in caregiver questionnaire.

Construct	Measure	Items/description of measure	Response options	Reliability/validity
Demographics	Purpose-designed items	Parent demographics (e.g. highest level of education)	Various	N/A
		Child demographics and medical characteristics (e.g. date of diagnosis)		
		‘How many times has your child been hospitalised in the previous 12 months?’	Numerical	
		‘How many days has your child been hospitalised in the previous 12 months?’	Numerical	
		For parents of children aged 5 years or older: ‘how many days of school has your child missed in the previous month?’	Numerical	
Distress	Kessler Psychological Distress Scale (K10)[22]	Parents rated how often they experienced 10-items (e.g. ‘Nervous’, ‘Hopeless’, and ‘So depressed that nothing could cheer you up’) over the previous 30 days.	5-Point Likert scale from 1 (none of the time) to 5 (all of the time)	• The K10 has strong predictive validity for clinically relevant distress and can be compared to population norms^[18, 22, 23]^. • Our sample showed that the K10 had strong internal reliability (Cronbach’s α = .90).
Perceived health of child with chronic illness and impact	Purpose-designed VAS items	‘Rate your child’s overall health’	0 (worst health/no burden/not at all/no impact) to 100 (best health/extreme burden/extremely well/extreme impact)	• These items were inspired by the EQ-VAS – a global health rating visual analogue scale^[24]^. • Visual analogue scales (VASs) are useful in collecting measurements with more variability^[25]^. They have been shown feasible to implement and to have adequate sensitivity across several constructs such as depression and pain^[26, 27]^.
		‘Rate how much your child’s medical condition or treatment affects their activity levels’		
		‘Rate how much your child worries about their own health’		
		‘Rate how well you believe you are coping with your child’s condition and treatments’		
		‘Rate how much your child’s condition and treatments affect your own mental health and wellbeing’		
		‘Describe what would help to feel more supported, if anything’	Open-ended	

#### K10 measure of psychological distress

As per the K10 instructions, we calculated a total score by summing all responses. Total scores range from 10 to 50, with higher scores indicating higher distress. We grouped scores into four levels of distress: low distress (total score = 10–15), moderate distress (total score = 16–21), high distress (total score = 22–29), and very high distress (total score = 30–50) (Australian Bureau of Statistics, 2012; NSW Government, 2020).

#### Visual analogue scales

We also used five purpose-deigned VAS to measure how parents perceived: (i) their child’s overall health (*0 = worst health, 100 = best health*); (ii) how much their child’s medical condition or treatments affect their activity levels *(0 = no burden, 100 = extreme burden);* (iii) their child worries about their own health *(0 = no burden, 100 = extreme burden);* (iv) they were coping with their child’s condition and treatments *(0 = not at all, 100 = extremely well);* and (v) their child’s condition and treatments affect the parent’s mental health and wellbeing *(0 = no impact, 100 = extreme impact)*.

#### Open-ended item

In a single open-ended question, we also asked parents to describe what would help them feel more supported, if anything.

### Data analysis

We used IBM SPSS Version 26 to conduct our quantitative analyses (IBM Corp, 2019). Four participants missed 1 of the 10 items on the K10. We imputed missing values using the mean substitution method (NSW Government, 2020; Sax Institute by the Priority Research Centre for Gender, 2010). Missing data for outcome variables were missing at random (Little’s MCAR test: χ^2^(138) = 159.472, *p* = .102).

We used descriptive statistics to present the socio-demographics. We used bivariate Pearson’s Correlation to determine the relationship between overall distress scores and VAS items. We accounted for any non-independence by using linear mixed-effects models with random intercepts for each family to assess how distress scores and VAS items differed between the illness groups. We compared main effects for each illness group using the Sidak confidence interval adjustment to counteract the multiple pairwise comparisons.

We used NVivo to conduct a qualitative content analysis of participants’ responses to the single open-ended question (QSR International Pty Ltd, 2018). We followed Elo and Kyngas’ guidelines (Elo and Kyngäs, 2008) for content analysis which involved preparation, organisation, and reporting the data. In the preparation phase, the lead researcher (LK) read participants’ responses multiple times to become familiar with the data. In the organisation phase, LK began coding responses line-by-line and organised codes into a coding tree. RI then coded 10% of participants’ responses (Burla et al., 2008; Campbell et al., 2013). Both LK and RI then organised codes into overarching categories and compared categories across the five illness groups to determine whether participants’ responses differed according to their child’s illness. In the reporting phase, we describe our interpretation of the categories and provide illustrative quotes with pseudonyms for each participant.

## Results

### Participants

We sent out 285 questionnaires, from which we received 151 completed questionnaires (response rate = 53.0%). Of the 151 completed questionnaires, 114 (75.5%) were completed by a parent of a child with cancer, CF, kidney disease, gastrointestinal disorder, or DEE. Seven parents reported having two children with two different diagnoses; these parents were excluded from analysis to allow for accurate cross-group comparisons. Due to largely missing data for one parent, we present on the results of 106 parents from across the 5 included diagnoses.

Most parents who completed our survey were mothers (*n* = 79, 74.5%), with English as their first language (*n* = 92, 86.8%), employed at least part-time (*n* = 83, 78.3%), and university-level educated (*n* = 67, 63.3%). Over half of the families had two children (*n* = 62, 58.5%). Most parents had a child diagnosed with cancer (*n* = 48, 45.3%), followed by CF (*n* = 27, 25.5%). The median child age at survey completion was 8.0 years (*interquartile range [IQR]* = 5.0, 12.0 years), and children were a median of 5.0 years from diagnosis (*IQR* = 2.0, 11.0 years). Socio-demographics are detailed in Table 2.

**Table 2.** Socio-demographics of participating caregivers, and medical characteristics of their children (*N* = 106).

Parent demographics	Median (IQR)/*n* (%)
Parent age	42.0 years (38.0–47.0 years)
Mother/father/‘other’	79 (74.5%)/27 (25.5%)/0 (0%)
First language (missing = 2, 1.9%)	
English	92 (86.8%)
Other	12 (11.3%)
Highest education	
Year 11 or below	4 (3.8%)
Year 12	9 (8.5%)
Certificate/diploma	22 (20.8%)
University degree	45 (42.5%)
Apprenticeship	4 (3.8%)
Postgraduate degree	22 (20.8%)
Employment	
Full-time	43 (40.6%)
Part-time	40 (37.7%)
Casual	6 (5.7%)
Not employed	
Home duties	13 (12.3%)
Not seeking work/retired/actively seeking	4 (3.7%)
Annual household income before tax (missing = 1, 0.9%)	
Nil	1 (0.9%)
Less than $29,999	1 (0.9%)
$30,000–$59,999	5 (4.7%)
$60,000–$89,999	4 (3.8%)
$90,000–$119,999	11 (10.4%)
Greater than $120,000	54 (50.9%)
Prefer not to answer	29 (27.4%)
Number of children per family	
2 Children	62 (58.5%)
3 Children	39 (36.8%)
4 Children	5 (4.7%)
Number of children with a chronic illness per family	
1 Child	105 (99.1%)
2 Children^a^	1 (0.9%)
Child demographics	Median (IQR)/*n* (%)
Age of child at survey completion	8.0 years (5.0–12.0 years)
Time since diagnosis (missing = 4)	5.0 years (2.0–11.0 years)
Female child/male child (missing = 1, 0.9%)	53 (50.0%)/52 (49.1%)
Primary diagnosis	
Cancer	48 (45.3%)
Developmental and epileptic encephalopathy	10 (9.4%)
Kidney disease	12 (11.3%)
Gastrointestinal disorder	9 (8.5%)
Cystic fibrosis	27 (25.5%)
Hospitalisations in the past year	1.0 hospitalisations (0–3.25 hospitalisations)
Days in hospital in the past year (missing = 1)	6.0 days (0–25.5 days)
Days of school missed in the past month^b^ (missing = 3)	2 days (0–6.75 days)

IQR: interquartile range.

^a^This parent was included in analysis as both children were diagnosed with CF.

^b^For parents of children 5 years or older (*n* = 87).

#### Hospitalisations and missed school

The number of times participants’ children had been admitted to hospital differed between groups (*F*(4,91) = 2.56, *p* = .044). However, pairwise comparisons did not reveal significant differences. The number of days children had spent in hospital over the previous year differed between groups (*F*(4,85) = 5.27, *p* = .001) – children with cancer spent more days in hospital in the previous year than children with CF (*mean difference[MD]* = 39.85, 95% CI [9.65, 70.06]) or DEE (*MD* = 42.90, 95% CI [0.89, 84.92]). Number of days of school missed over the previous month for children over 5 years of age did not differ between groups (*F*(4,65) = 1.24, *p* = .304).

#### Parent distress and wellbeing

**Figure 1.**
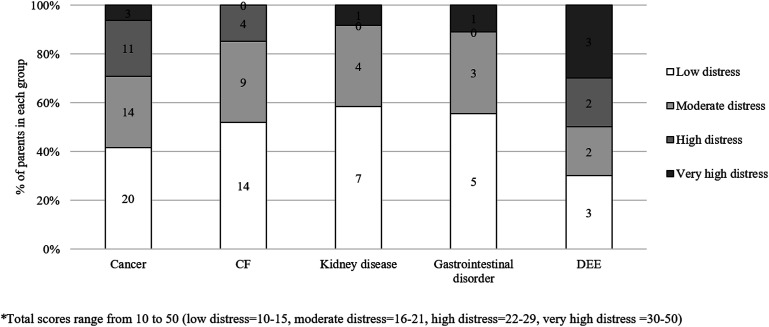
Parents’ distress scores, by health condition.

About half of the parents reported ‘low’ distress (*n* = 49, 46.2%) (see Figure 1). We found some evidence that total distress scores differed between groups (*F*(4,80) = 2.51, *p* = .049), with parents of children with DEE reporting higher distress than parents of children with CF (*MD* = 6.76, 95% CI [0.11, 13.42]). We found no other differences in total distress scores.

**Figure 2.**
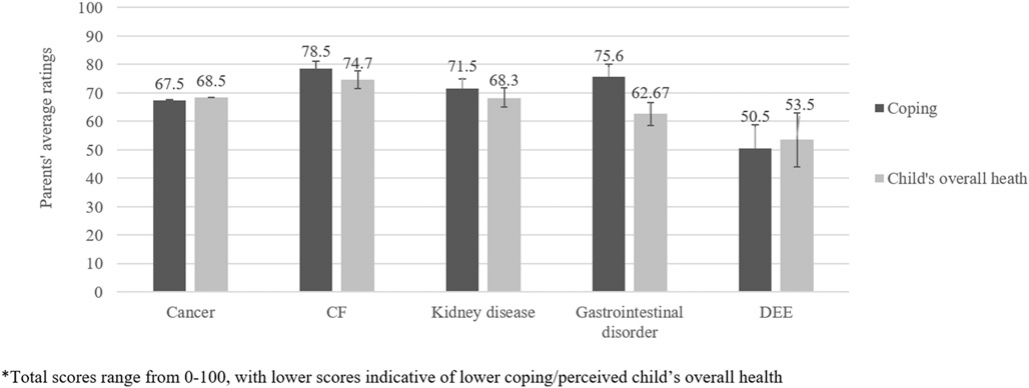
Parents’ average ratings for coping and child’s overall health, by health condition.

We found small-to-medium sized correlations between distress scores and parents ratings of their child’s health (*r*(104) = 0.340, *p* < .001, 95% CI [0.09, 0.53]), impact of medical condition/treatments on activity levels (*r*(100) = 0.233, *p* = .018, 95% CI [0.04, 0.40]), how well they are coping (r(103) = 0.536, *p* < .001, 95% CI [0.41, 0.67]), and how much their child’s health affects their own mental health and wellbeing (*r*(102) = 0.490, *p* < .001, 95% CI [0.36, 0.61]). Parents’ ratings of their coping with their child’s condition/treatments differed between illness groups (*F*(4,81) = 3.24, *p* = .016), but reports of the effect of their child’s condition and treatments on their own mental health and wellbeing did not (*F*(4,77) = 1.40, *p* = .244). Pairwise comparisons showed that parents of children with DEE rated their coping as poorer than parents of children with CF (*MD* = −25.32, 95% CI [−46.52, −4.11]) (see Figure 2).

#### Perceived health of child with chronic illness

Parents’ rating of their child’s overall health did not differ by illness group (*F*(4, 84) = 1.51, *p* = .208) nor the degree to which the affected child worried about their own health (*F*(4,73) = 1.87, *p* = .125) (see Figure 2). However, parent-reported impact of their child’s medical condition/treatments on children’s activity levels differed between groups (*F*(4,81) = 4.68, *p* = .002). Pairwise comparisons showed that parents of children with DEE reported greater effects on activity levels than CF (*MD =* 39.28, 95% CI [6.72, 71.83]), and parents of children with cancer reported greater effects on activity levels than CF (*MD =* 28.55, 95% CI [6.53, 50.57]).

#### Parent unmet support needs

Fifty-seven parents (53.8%) reported having at least one unmet support need in the open-ended question, which we categorised into key themes.

##### Mental health support

Parents reported needing more mental health support for the whole family to facilitate family functioning (*n* = 10), mental health support for siblings (*n* = 12), and mental health support for parents (*n* = 4). We identified one illness-specific sub-theme, with parents of children with cancer commenting on the mental health support gap for their family when their child had completed treatment.

‘With families continuously feeling pulled apart due to the needs of their chronically ill child there needs to be more support for the families and their children, to find ways to help support these families is essential for the wellbeing of the entire family. From personal experience it always feels like one parent is caring for the chronically ill child and the other is caring for the siblings, always feeling like the family is being pulled in different directions and not functioning as a whole family unit’ – Sarah, a mother with a child with a DEE.

‘I feel there is limited mental health support once you leave the hospital environment for “survivors”’ – Jade, a mother with child with cancer.

##### Practical and financial support

Parents reported needing practical support in their home to help with their children – both the ill child and healthy siblings (*n* = 11). This included support with childcare, meal preparation, and at-home medical care for their ill child. Four parents of children with CF specifically reported needing more help with their child’s physiotherapy. All 11 parents reported not being able to afford specialists or carers to provide support them in their child’s day-to-day care. These parents described needing to undertake all caring duties themselves, which meant they were either unable to work fulltime or at all. Parents acknowledged that this exacerbated their financial distress. Eight parents stated they needed further government financial support and lamented the fact that their child was deemed ineligible for government disability payments.

‘Emotionally I am well-supported however physically our family would benefit from access to physio in the home and meals provided to the caregiver when our son has hospital admissions’ – Angela, mother with a child with cystic fibrosis.

‘Part of the issue for me is the invisibility of caring for someone with a chronic disease. It is a job that requires advanced level caring, with no particular training, is not recognised and not compensated’ – Tanya, mother with a child with cancer.

##### Community support

Ten parents reported the importance of being able to speak with other parents with a child diagnosed with the same illness and/or who understood their experiences. Parents discussed the support derived from other parents in the hospital and in private social media groups. Parents reported wanting more consistent opportunities to meet with other parents, particularly for face-to-face meetings. For gastrointestinal disorders, three parents wanted greater community awareness of their child’s conditions. These parents discussed challenges they face with limited community understanding of the impact of their child’s condition on the child and family.

‘There needs to be more awareness of chronic illnesses that don’t fit in the normal “box” of what’s considered a chronic illness. My daughter suffers from severe [gastrointestinal disorders]. For the last two years she’s only slept 2–3 hours a day due to the discomfort of her symptoms. Disrupted sleep and hearing loss has led to a range of developmental delays. We alternate between appointments at her specialists and the therapies she has to attend. It takes up all of our time leaving no time for social or learning activities normal kids her age can engage with’ – Bianca, mother with a child with a gastrointestinal disorder.

##### Health services support

Six parents reported wanting more frequent and longer communication with their child’s healthcare professionals. These parents reported feeling rushed during clinic appointments or when the specialists would visit their child on the ward. Six parents also reported wanting more verified information that is relevant to their child’s specific illness. Parents particularly wanted this information to be provided in writing and from the point of diagnosis. They also wanted this information to be provided by their child’s healthcare professionals, as opposed to searching for information themselves on online.

‘When your child is first diagnosed there is not enough information provided to the parents that really explains what the illness is and how to manage it. I also think there is a lot of assumed knowledge when we have clinic visits… It would be good if there was a lot more information written down that could be accessed at your convenience’ – Kate, mother with a child with cystic fibrosis.

Three parents reported wanting an ‘advocate’ for their child. They wanted this advocate to be a central point of contact within the hospital system, who could distil and simplify the information provided by all their child’s treating specialists.

‘I think there should be more patient advocates available to families to help navigate the medical system and government support. Specialists can be difficult to understand and are time poor so someone who can translate medical speak. Maybe family meetings with an external and experienced person who can explain what is going on, help define people’s roles within the family, answer specific questions’ – Lisa, mother with a child with a DEE.

##### School support

Nine parents reported wanting greater school support for their child with chronic illness. Parents wanted schools to be more accommodating of their child’s condition, and provide greater flexibility around attendance, completion of assignments, and participation with various activities. Three parents noted the academic toll that their child’s chronic illness had had on their other children. These parents reported wanting schools to be more accommodating and supportive of siblings, acknowledging the challenges that many siblings face.

‘Siblings do at times get forgotten. In our case our third child suffered academically. I wished there was warning and some netting to catch him. We are doing catch up now. Emotionally he had many around him but schooling was tough’ – Emily, mother with a child with cancer.

## Discussion

We aimed to determine how parents report their own distress, and their own and their child’s wellbeing, and whether this differs across illness groups. We found that many parents reported low psychological distress and that they were coping well, with small-moderate correlations between these variables. While parents’ rating of their child’s overall health did not differ by illness group, we found that parents with a child with DEE appeared to be faring the worst regarding their psychological distress and perceived coping, especially when compared to parents of children with CF. We also aimed to explore how parents describe their support needs and whether these differ across illness groups. Parents in our study reported a range of unmet mental health, practical and financial, community, health services, and school support needs for all family members.

Quantitatively, parents with a child with a DEE appeared to be faring the worst. These parents reported the highest average psychological distress across the five illness groups, which was statistically more than reported in parents of children with cystic fibrosis, as well as reporting lower perceived coping than parents of children with cystic fibrosis, kidney disease, or a gastrointestinal disorder. Many studies have reported a negative impact of DEEs on parents’ wellbeing, with quality of life lower than population norms (Gallop et al., 2021b; Robertson et al., 2024). Previous research measuring distress (via the K10) in parents with a child with cancer showed a lower proportion of ‘high’ distress than seen with our DEE parents (McCarthy et al., 2016). The added challenges of DEE, such as developmental regression, and cognitive impairment may compound the psychosocial challenges that these families face (Gallop et al., 2021a; Nevin et al., 2022; Spratt et al., 2007). Indeed, distress (measured via the K10) in parents who have a child with physical and intellectual disability appears to align more closely to what we saw in our DEE sample. Poorer overall health of a child with DEE (or at least, perceptions of poorer overall health) and limitations to the child’s activity, as reported in our study, may contribute to parents’ higher levels of psychological distress.

Interestingly, in our small sample, parents across illness groups did not differ in perceptions of how much their child’s condition and treatments impacted their own mental health and wellbeing. This is despite the differences we found in our samples’ distress scores and perceived coping for the DEE group versus CF group. The ‘Common-Sense Model of Self-Regulation’ proposes that individual’s perceptions of illness influence their coping, which in term influences their wellbeing (Leventhal et al., 2016). Research has highlighted the impact of illness perceptions on coping in parents who have a child with a neurological condition, and the relationship between illness perceptions and psychological wellbeing across a range of illnesses (Dempster et al., 2015). Further investigation is therefore needed to discern whether this is a real null-effect, with coping in our sample potentially playing a different role than in other illnesses, or whether we lacked adequate statistical power.

Qualitatively, parents in our study reported a range of unmet support needs. Consistent with previous research (Pelentsov et al., 2015), parents in our study commonly reported a need for greater mental health support for their family. Psychosocial support for family members is extremely important in chronic conditions, particularly given the bi-directional relationship between parent functioning and their child’s adaptation to illness (Eccleston et al., 2015; Law et al., 2019). The psychosocial health of siblings of children with chronic illness is also significantly lower than that of healthy controls (Dinleyici et al., 2020), with few available wellbeing interventions dedicated to siblings (McKenzie Smith et al., 2018). This is despite the evidence supporting the potential for sibling-focused interventions in enhancing emotional and behavioural outcomes (Hartling et al., 2014). A critical challenge in better addressing the wider family unit is that, within most hospital settings, siblings and parents are not allocated a medical record number. This limits their access to hospital-based services, relying on greater integration with community services.

With little access to hospital-based services, parents in our study emphasised the value of building a strong peer-support network. This is consistent with previous research which has found that peer networks can reduce psychological distress, improve ability to cope, and provide altruistic benefit in supporting others (Baumbusch et al., 2018; Bray et al., 2017). Research also shows that parents seeking wellbeing support tend to gravitate toward peer-support strategies as they help to reduce feelings of isolation (Oakley et al., 2022). Greater access to peer-support networks, particularly facilitated by non-profit, community-based organisations, may be needed for disease groups such as DEE or gastrointestinal disorders where poorer coping or a lack of community awareness was reported by the parents in this study. In rarer diseases, having greater peer-support may also alleviate burden on clinicians, given parents’ role as ‘expert caregiver’ (Baumbusch et al., 2018).

Practical and financial support were also key unmet needs in our study. More than half of the parents in our study reported higher than average Australian household income ($2,086AU a week) (Australian Bureau of Statistics, 2020), and yet many still reported not being able to afford specialists or skilled carers. Research highlights that child chronic illness impacts parental employment, particularly for mothers (Hatzmann et al., 2014; Kish et al., 2018). Similarly, parents of children with cancer have reported multiple sources of financial toxicity in addition to reduced working hours (e.g. costs incurred in travelling for treatment) in the years following treatment completion (Kelada et al., 2020b). Research in kidney disease has shown that parent-rated child health was worse in families with lower household income (Didsbury et al., 2019). Issues of financial wellbeing or strain relate to health equity. Addressing financial needs is integral to a public health approach that recognises the family unit within childhood chronic illness (Gardiner et al., 2020).

### Study limitations

To our knowledge, our study is one of the first to compare parent psychological distress and wellbeing across several serious chronic illness groups. It also begins to address the limited research on parental wellbeing in more rare, chronic conditions. However, our study is not without limitations. Design wise, these data were collected as part of a larger study examining sibling wellbeing. This may have resulted in biased respondents, with the potential for families who required further support for healthy siblings more likely to participate. As with many psychosocial research studies (Koumarianou et al., 2021), our sample was also predominantly mothers, of a high education, from an English-speaking background, and with a higher-than-average income. Further investigation is needed to address the experiences of culturally and linguistically diverse parents with a child with a chronic illness, as well as the needs of parents who have more than one child with multiple diagnoses.

Statistically, we had a relatively small sample size, with an uneven representation across illnesses, and did not take into account the severity of each illness. Our pairwise analyses allowed us to identify potential greater challenges for parents who have a child with DEE; however, the multiple analyses may have increased risk of Type 1 errors. While the K10 is well-validated and VAS is widely used, our VAS question stems were purpose-designed.

### Implications for practice

Further practical and financial support is needed for parents of children with chronic illness, alongside mental health support which accounts for the time and energy-consuming role of caretaking. Exploration into the relationship between parents’ illness perceptions and wellbeing is warranted. We hypothesise that interventions to improve illness perceptions may be beneficial in improving caregiver wellbeing. Parents in the current study suggested that interventions which facilitate peer support may be particularly beneficial in their coping, and this has also been supported by previous research (Oakley et al., 2022). Interventions which balance feasibility and provide a clinically relevant ‘dose’ require examination. Healthcare professionals can also help by acknowledging the impact of a serious chronic illness on caregivers. Facilitating discussions around parents’ mental health by actively inquiring about how parents are coping and creating an environment where parents feel safe to make mental health disclosures may be beneficial (Franklin et al., 2021). Similarly, parents in our study emphasised the need for further support for the healthy siblings. Sustainable and co-designed sibling support is needed to help bolster siblings’ coping skills (Kelada et al., 2021; Thabrew et al., 2018). Gold-standard consumer involvement throughout development and reimbursement for participation may enhance engagement in such interventions.

### Conclusion

Our study begins to fill the knowledge gap regarding psychological wellbeing and support needs among parents of children across different serious chronic illness groups. We found there were differences across illnesses –in terms of parent distress, coping, and support needs. This suggests that support interventions may be most effective if they are tailored to the child’s illness, with greater support potentially needed for parents who have a child with a DEE.
